# Effective combination gene therapy using CEACAM6-shRNA and the fusion suicide gene yCDglyTK for pancreatic carcinoma *in vitro*

**DOI:** 10.3892/etm.2012.774

**Published:** 2012-10-30

**Authors:** HONGYU LONG, QINGFU LI, YUAN WANG, QIAN LI, TING LIU, JIE PENG

**Affiliations:** Department of Gastroenterology, Xiangya Hospital, Central South University, Changsha, Hunan 410008, P.R. China

**Keywords:** pancreatic carcinoma, RNA interference, suicide gene therapy, carcinoembryonic antigen-related cell adhesion molecule

## Abstract

The incidence of pancreatic carcinoma, a gastrointestinal malignancy, is on the increase and effective therapeutic strategies are therefore required. This study aimed to construct a recombinant plasmid pcDNA3.1(-) shCEACAM6-yCDglyTK from CEACAM6 targeting shRNA and the fusion suicide gene yCDglyTK for inhibition of SW1990 human pancreatic carcinoma cell growth and invasion. A plasmid containing hU6 promoter and CEACAM6 targeting short hairpin RNA (CEACAM6-shRNA) frame was constructed. It was subcloned to a CEA promoter-driven fusion suicide gene pcDNA3.1(-)yCDglyTK. The recombinant plasmid pcDNA3.1(-) shCEACAM6-yCDglyTK was identified by restriction endonuclease analysis and DNA sequencing. The recombinant plasmid was delivered into SW1990 human pancreatic carcinoma cells, the mRNA and protein expression of yCDglyTK and CEACAM6 was examined by RT-PCR, western blot analysis and immunofluorescence. SW1990 cells were treated with the prodrug 5-fluorocytosine (5-FC), and the cell viability was evaluated using the 3-[4,5-dimethylthiazol-2yl]-2,5-diphenyl tetrazolium bromide (MTT) assay. The invasiveness and migration of SW1990 cells were evaluated by transwell migration assays. The restriction endonuclease analysis and DNA sequencing confirmed the construction of the recombinant plasmid pcDNA3.1(-) shCEACAM6-yCDglyTK. Reverse transcription polymerase chain reaction (RT-PCR) and western blot analysis outcomes showed that yCDglyTK was expressed in SW1990 cells and expression of CEACAM6 in SW1990 cells was significantly knocked down. MTT assay showed that the mean viability of SW1990 cells was significantly reduced after administration of the prodrug 5-FC *in vitro*. Transwell migration assays showed that invasion and migration action of SW1990 cells was significantly inhibited. In conclusion, recombinant plasmid pcDNA3.1(-) shCEACAM6-yCDglyTK was successfully constructed. The recombinant plasmid may therefore serve as a novel gene therapy approach for pancreatic carcinoma.

## Introduction

Pancreatic carcinoma is one of the most frequently occurring gastrointestinal malignancies and the incidence rate has shown an upward trend worldwide (1). The prognosis for patients with advanced pancreatic carcinoma remains poor with a 5-year survival rate of <5% (1). Among the most significant determinants of the poor prognosis associated with this malignancy are the highly aggressive loco-regional invasion and early metastasis that characterise this malignancy, such that the majority of patients present with advanced, surgically unresectable disease (2). Gemcitabine and erlotinib are the only agents that are approved for the treatment of pancreatic carcinoma. However, both drugs induce a poor response in patients and their use can result in patients developing multiple drug resistance (3,4). Although in recent years, great progress has been observed with regard to investigations on the molecular pathogenesis of pancreatic carcinoma, the clinical treatment of pancreatic carcinoma remains a challenge. Therefore, novel therapeutic approaches to this malignancy are needed.

Gene-direct enzyme/prodrug therapy (GEPT), or suicide gene therapy, aims to improve the therapeutic efficacy of conventional cancer radio- and chemotherapy without side-effects (5,6). This system has received a great deal of attention for its clinical and therapeutic potential to treat cancer. At present, a large number of enzyme/prodrug systems have been developed for GEPT, two of which are the herpes simplex virus thymidine kinase/ganciclovir (HSV-TK/GCV) and cytosine deaminase/5-fluorocytosine (CD/5-FC) (7). Recently, some studies (8,9) have tried to enhance the therapeutic effect of suicide gene therapy by combining it with other gene therapies. However, the combination of fusion suicide gene therapy with anti-angiogenesis gene therapy for pancreatic carcinoma has yet to be reported.

Carcinoembryonic antigen-related cell adhesion molecule (CEACAM)6, also known as CD66c or NCA-90, as well as another 6 members of the CEACAM subgroup, belong to the human carcinoembryonic antigen (CEA) family (10). CEACAM6 overexpression was found in a wide variety of epithelial cancer types such as lung, breast, colorectal, and hepatocellular carcinomas (11–14). CEACAM6 has also become a target for pancreatic cancer therapy (15,16). Overexpression of CEACAM6 was found in >90% of invasive pancreatic adenocarcinomas (16). RNA interference (RNAi) is a process involving sequence-specific and post-transcriptional gene silencing. CEACAM6-specific RNAi decreases cancer cell proliferation, metastasis and angiogenesis in pancreatic cancer (15).

In the present study, we aimed to test the feasibility of a novel therapeutic vector system involving a combination of suicide gene therapy and antiangiogenesis gene therapy. The *in vitro* experiments on pancreatic carcinoma SW1990 cells were studied using a triple-gene vector expressing CEACAM6-shRNA and the fusion suicide gene yCDglyTK.

## Materials and methods

### Cell lines and cell culture

The SW1990 human pancreatic carcinoma cell line (CEA positive) was obtained from the Cancer Research Institution, Central South University (Hunan, China). Cells were cultured in RPMI-1640 medium (Invitrogen Inc., Carlsbad, CA, USA) with 10% fetal bovine serum at 37°C in a humidified atmosphere of 5% CO_2_ and 95% air.

### Construction of the triple-gene plasmid of pcDNA3.1(-) shCEACAM6-yCDglyTK

pcDNA3.1(-)CV-yCDglyTK was constructed in our previous study (17). Expression of the fusion suicide gene yCDglyTK was regulated by CMV- enhanced CEA promoter and was expressed specifically in CEA-positive cells. Oligonucleotides encoding the corresponding small hairpin RNA (termed CEACAM6-shRNA) was generated by ligation of inserts targeting the following sequences into a hU6 promoter-contained pUC57-simple plasmid: 5′-CCG GACAGTTCCATGTATATTCAAGACGTATACATGGAAC TGTCGTTTTTT-3′ (sense: CCGGACAGTTCCATGTATA; loop: TTCAAGACG; antisense: TATACATGGAACTGT CCGG; termination code: TTTTTT). shCEACAM6 and pcDNA3.1(-)yCDglyTK were then fused by *Nhe*I and *Xba*I restriction endonuclease overnight at 37°C to form a recombinant plasmid of shCEACAM6 and the fusion suicide gene yCDglyTK .Thus, a novel triple-gene vector pcDNA3.1(*-*) shCEACAM6-yCDglyTK was developed. Double enzyme cutting and sequencing were performed to prove the accuracy of the new plasmid.

### Stable transfection in vitro

The novel triple-gene vector, pcDNA3.1(-)shCEACAM6-yCDglyTK plasmid was mixed with Lipofectamine 2000 at the combination rate of 1/2 to 1/3. SW1990 human pancreatic carcinoma cells were seeded in 6-well plates at a density of 2×10^5^ cells per well. When the cell monolayer reached 70–80% confluence, pcDNA3.1(-) null, pcDNA3.1(-)yCDglyTK and pcDNA3.1(-)shCEACAM6-yCDglyTK were added to different 6-well plates. The following day, a 1:10 passage of the transfected SW1990 cells was performed, followed by G418 selection (400 *μ*g/ml). Approximately 3 weeks later, the resistant colonies were picked and transferred to 96-well plates. These clones were maintained in selective culture medium (with 200 *μ*g/ml of G418). Surviving colonies transfected with pcDNA3.1(-)null, pcDNA3.1(-) yCDglyTK, or pcDNA3.1(-)shCEACAM6-yCDglyTK were designated as SW/null, SW/CDTK, or SW/shCEACAM6-CDTK, respectively, and subjected to further study.

### Reverse transcription polymerase chain reaction (RT-PCR) and western blot analysis

Total RNA from parental SW1990 cells and three different transfected cells was extracted using TRIzol reagent (Invitrogen Inc.). The quantity and quality of RNA was assessed by absorbance at 260 and 280 nm, respectively. The RT reaction was carried out using the ReverTra Ace^®^ RT Kit (Toyobo Co., Ltd., Osaka, Japan) as per the manufacturer’s instructions. Subsequently, we performed PCR on the cDNA product. For yCDglyTK, a PCR product of 707 bp was produced by the forward primer 5′-GGGAGATT AGAGGGCAAAGTGT-3′ and reverse primer 5′-ACGGCGT CGGTCACGGCATAA-3′. For CEACAM6, a PCR product of 356 bp was produced by the forward primer 5′-CGTTCAAT GTCGCAGAGGG-3′ and reverse primer 5′-CGCTGAGTA GAGTGAGGGT-3′. β-actin was used as an internal control and a PCR product of 285 bp was produced by the forward primer 5′-AGCGAGCATCCCCCAAAGTT-3′ and reverse primer 5′-GGGCACGAAGGCTCATCATT-3′.

For western blot analysis, cells were collected 72 h after transfection and lysed in loading buffer (20 mmol/l Tris-HCl, pH 7.5; 150 mmol/l NaCl, 1 mmol/l EDTA, 5 mmol/l DTT, 1% Triton X-100). The lysates were centrifuged at 12,000 × g for 15 min at 4°C. The supernatant was collected and protein concentrations were determined by the BCA protein assay. Protein (40 *μ*g) was separated via 15% SDS-PAGE and transferred to PVDF film (Amersham Pharmacia Biotech, Piscataway, NJ, USA). The films were incubated in blocking solution, consisting of 5% skimmed milk in TBS-T [10 mM Tris-HCl (pH 8.0), 150 mM NaCl and 0.1% Tween-20], for 1 h at room temperature, then probed with mouse anti-CEACAM6 antibody (Santa Cruz Biotechnology, Inc., Santa Cruz, CA, USA), rabbit anti-TK antibody (QED Bioscience, Inc., San Diego, CA, USA) or mouse anti-β-actin antibody (Sigma-Aldrich, St. Louis, MO, USA). This was followed by incubation with their respective peroxidase-conjugated secondary antibodies. Hybridization was visualized using the ECL chemiluminescence detection system (Kodak).

### CDTK/5-FC, shCEACAM6-CDTK/5-FC system-induced cytotoxicity

SW1990 cells (transfected and untransfected) were seeded in 96-well plates at a density of 8,000 cells per well and incubated at 37°C and 5% CO_2_ in humidified air for 24 h. The following day, 5-fluorocytosine (5-FC) was added into the culture medium at a final concentration of 200 *μ*g/ml. MTT assays were conducted at 24-, 48-, 72- and 96-h incubation time points to analyze cell viability. Twenty microliters of MTT solution (5 mg/ml, Sigma-Aldrich) were added to each well and incubated for 4 h. Dimethylsulfoxide (DMSO, Promega Corporation, Madison, WI, USA) was added to dissolve the blue crystal. The optical density (OD) was then determined using a multi-well plate reader (Awareness, model Stat-Fax-2100, USA) by measuring absorbance at 570 nm (OD570), with the absorbance at 690 nm as reference. The background absorbance of medium was also subtracted. Samples were assayed in triplicate, and the mean for each experiment was calculated. Cell growth curves were plotted, with culture time on the horizontal axis and OD570 on the vertical axis.

### Invasion and migration assay

The invasion assay was performed using an 8 *μ*m pore size transwell chamber in 24-well plates (Corning Costar, Cambridge, MA, USA). SW1990 cells stably expressing pcDNA3.1(-), pcDNA3.1(-)yCDglyTK, pcDNA3.1(-) shCEACAM6-yCDglyTK or untransfected SW1900 cells in 500 *μ*l of serum-free MEM medium were loaded into the top chamber with fetal bovine serum placed in the bottom chamber as a chemoattractant. After further incubation at 37°C for 10 h, the cells on the top of the filters were removed with cotton swabs. The cells on the lower surface of the filters were fixed in 4% paraformaldehyde and stained with 0.1% crystal violet. The crystal violet was removed and the cells were washed three times with phosphate-buffered saline (PBS). The remaining crystal violet staining of the migrated cells was eluted with one wash with 33% acetic acid. The OD540 nm of the eluted crystal violet was determined as a measure of migrated cells. Each experiment was performed in triplicate. The invasion of different groups was observed under a microscope. The cell migration assay was performed in a similar mode, except that cells were seeded into the uncoated filter and incubated for 24 h. Each measurement was performed in at least three independent experiments.

### Statistical analysis

Statistical analysis was performed by one-way analysis of variance (ANOVA) test. P<0.05 was considered to indicate a statistically significant difference. Numeric data were presented as the mean values ± standard deviation (SD).

## Results

### Recombinant plasmid was successfully constructed

**Figure 1:**
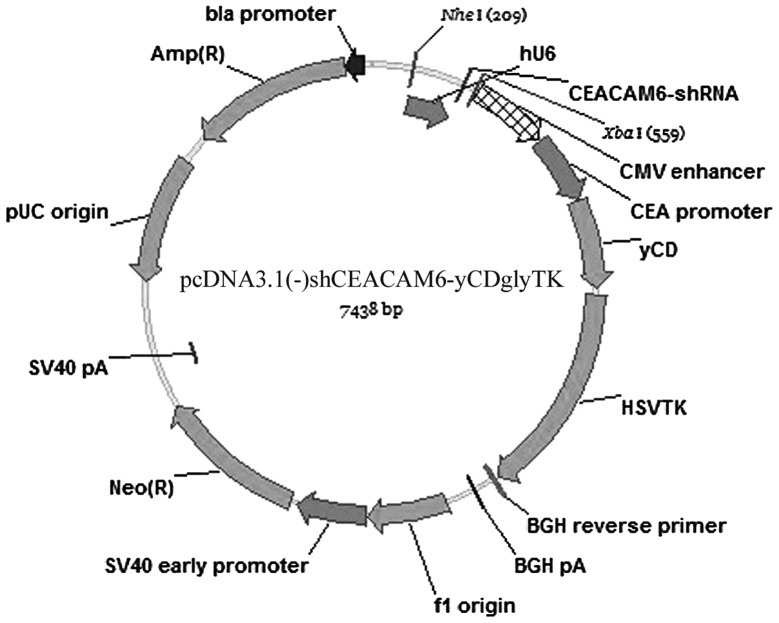
Plasmid profile of pcDNA3.1(-)shCEACAM6-yCDglyTK.

**Figure 2:**
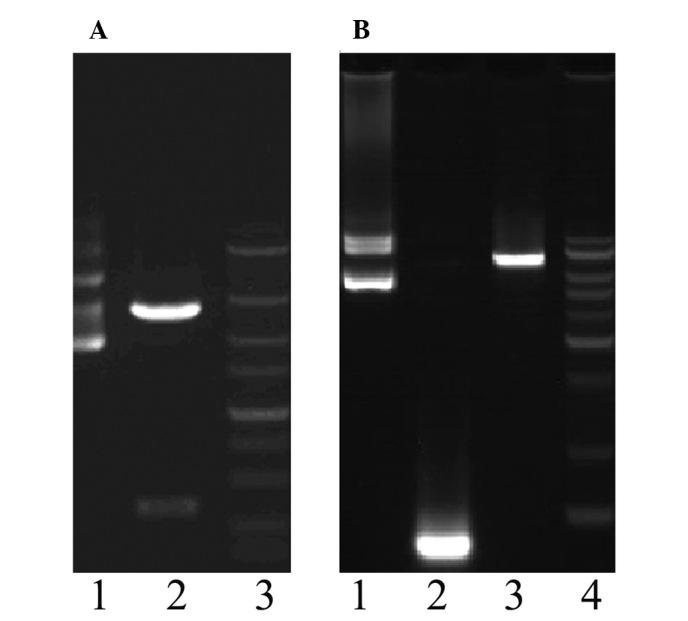
Identification for the constructed gene plasmid. (A) Enzyme cutting outcome of plasmid pcDNA3.1(-)CEACAM6-shRNA. Lane 1, pUC57-simple-hU6-CEACAM6-shRNA; lane 2, pUC57-simple-hU6-CEACAM6-shRNA by enzyme cutting; lane 3, DL5000. (B) Identification for pcDNA3.1(-) shCEACAM6-yCDglyTK; lane 1, pCDNA3.1(-)shCEACAM6-yCDglyTK; lane 2, PCR ladder; lane 3, pcDNA3.1(-)shCEACAM6-yCDglyTK by enzyme cutting; lane 4, KB ladder.

An interfering plasmid targeting CEACAM6 was initially constructed. The CEACAM6-shRNA expression cassette was subcloned into pcDNA3.1(-)CV-yCDglyTK to construct the novel vector pcDNA3.1(*-*)shCEACAM6-yCDglyTK (Fig. 1). In this novel triple-expressing plasmid, the CEACAM6-shRNA sequence was placed under control of the U6 promoter, while the fusion suicide gene yCDglyTK was driven by a CMV-enhanced CEA promoter. Newly constructed gene plasmid pcDNA3.1(-)shCEACAM6-yCDglyTK was identified by double-enzyme cutting and sequencing. As expected, the size of the newly constructed plasmid fragment by enzyme cutting was 7.5 kb (Fig. 2). Sequencing results showed that the newly constructed target-combined double suicide gene plasmid was in concordance with pcDNA3.1(-) shCEACAM6-yCDglyTK (data not shown).

### Recombinant plasmid was effectively delivered into SW1990 cells in vitro

**Figure 3:**
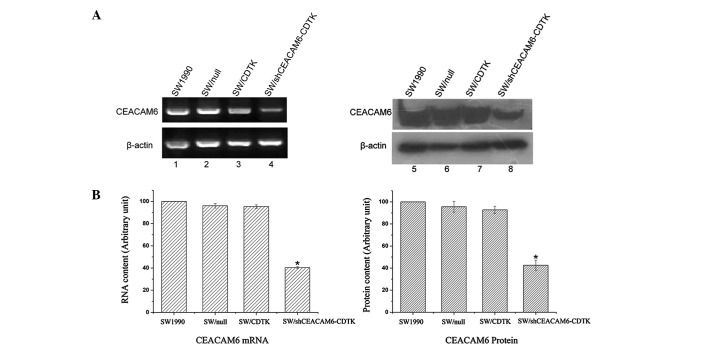
Inhibition of CEACAM6 mRNA and protein expression by transfection with pcDNA3.1(-)shCEACAM6-yCDglyTK. (A) Representative CEACAM6 mRNA and protein expression were analyzed by semiquantitative RT-PCR (left panel) and western blot analysis (right panel), respectively. β-actin was used as an internal control. RT-PCR: lane 1, parent SW1990; lane 2, SW/null; lane 3, SW/CDTK; lane 4, SW/shCEACAM6-CDTK. Western blot analysis (lane 5–8) followed the same sequence as RT-PCR. (B) The density of each band was measured, densities of CEACAM6 were normalized against corresponding β-actin signals, and relative intensities were expressed in arbitrary units where the intensity of parent SW1990 cells was set to 100%. The results are expressed as means ± standard deviation (SD) from three independent experiments.

**Figure 4:**
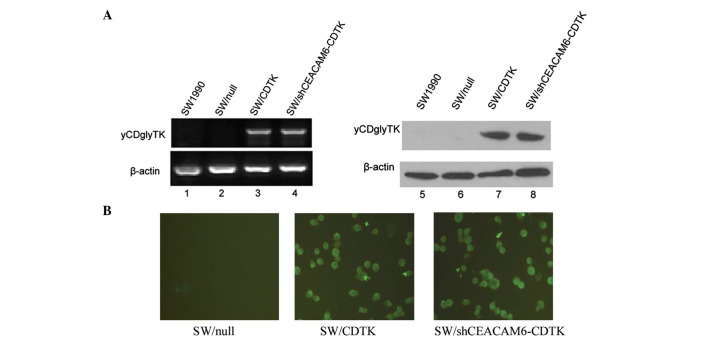
Expression of yCDglyTK by transfection with pcDNA3.1(-)shCEACAM6-yCDglyTK. (A) Representative yCDglyTK mRNA and protein expression were analyzed by semiquantitative RT-PCR (left panel) and western blot analysis (right panel), respectively. β-actin was used as an internal control. lane 1, parent SW1990; lane 2, SW/null; lane 3, SW/CDTK; lane 4, SW/shCEACAM6-CDTK. Western blot analysis (lane 5–8) followed the same sequence as RT-PCR. (B) Representative yCDglyTK protein expression detected by immunofluorescence assays. (original magnification, ×200).

This novel vector was delivered into SW1990 cells and stably transfected cell lines were obtained by G418 selection. At the same time, SW1990 cells stably transfected with three other plasmids, pcDNA3.1(-)null, pcDNA3.1(-) yCDglyTK and pcDNA3.1(-)shCEACAM6-yCDglyTK were also established. The expression of CEACAM6 and yCDglyTK were determined via RT-PCR, western blot analysis and immunofluorescence. Compared with parent SW1990 cells and SW/null, mRNA and protein levels of CEACAM6 were significantly decreased in SW/shCEACAM6-CDTK (Fig. 3). yCDglyTK was confirmed to be expressed in SW/CDTK and SW/shCEACAM6-CDTK cells, but not in the parent SW1990 cells and SW/null (Fig. 4).

### Recombinant plasmid pcDNA3.1(-)shCEACA M6 - yCDglyTK/5-FC system resulted in cytotoxicity in SW1990 cells

**Figure 5:**
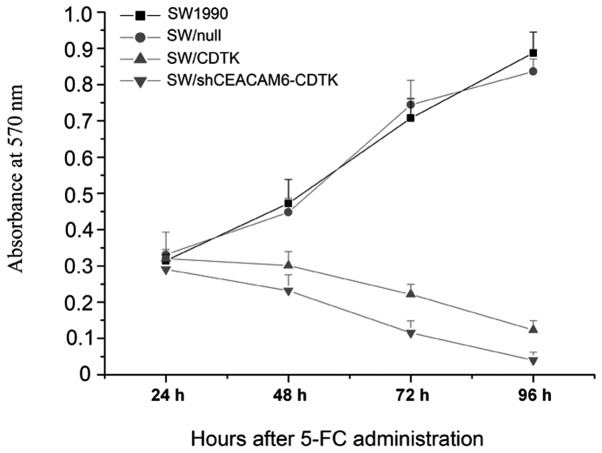
Growth curves of SW1990 cells and three different transfectants following the administration of 5-fluorocytosine (5-FC). SW1990 cells (transfected and untransfected) were maintained in culture medium containing 5-FC (200 *μ*g/ml). At the time points of 24, 48, 72 and 96 h, cells of each group were subjected to MTT assays. Cell growth curves were plotted, with culture time as the horizontal axis and OD570 as the vertical axis.

After a 24-h treatment with 5-FC, the OD570 of SW/CDTK cells and SW/shCEACAM6-CDTK cells decreased significantly compared to SW1990 or SW1990/null cells (P<0.01), as shown in the cell growth curve in Fig. 5. As time progressed, a high proliferation rate was maintained in untransfected SW1990 and SW/null cells. After 48 h, the OD570 did not increase in SW/CDTK or SW/shCEACAM6-CDTK cells. At 72- and 96-h treatment with 5-FC, a decrease was observed for the OD570, suggesting that most cells in the process of being killed. Low shCEACAM6-CDTK cell viability was detected.

### Recombinant plasmid pcDNA3.1(-)shCEACAM6-yCDglyTK inhibited SW1990 cell invasion and migration

**Figure 6:**
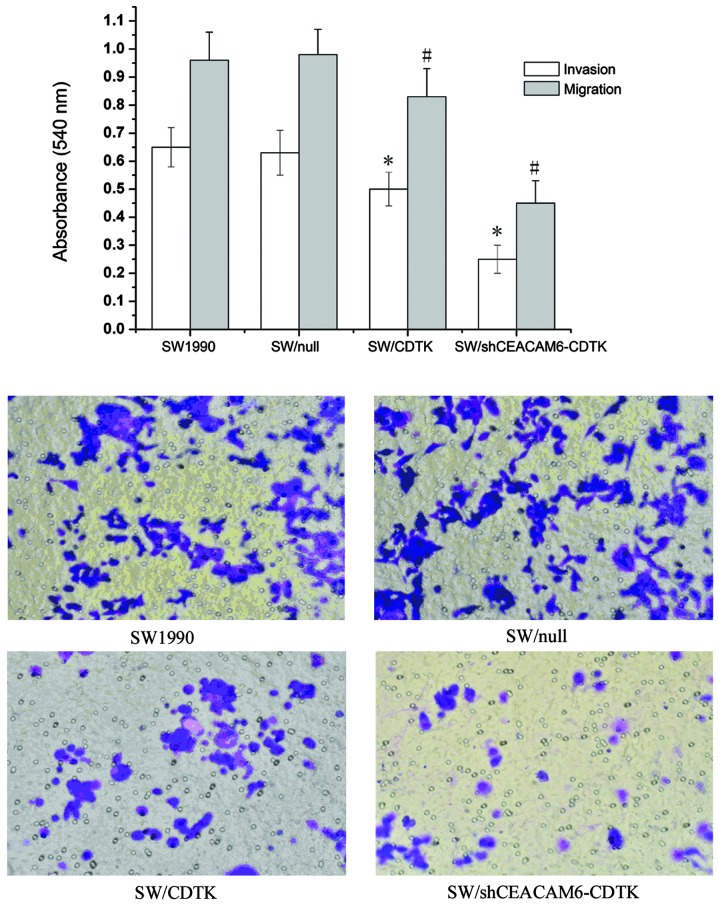
Invasion and migration assay. (A) Compared with SW1990 and SW/null, the cell invasiveness and migration of SW/CDTK and SW/shCEACAM6-CDTK were reduced. (B) The polycarbonate filters were stained with crystal violet and viewed under a light microscope (magnification, ×200). These experiments were performed three times.^*^Significant difference from SW1990 and SW/null groups in white bars, P<0.01; ^#^Significant difference from SW1990 and SW/null groups in grey bars, P<0.01.

The motility of three different transfected cells across transwell polycarbonate membranes was evaluated. As shown in Fig. 6A, compared with SW1990 and SW/null, the cell invasiveness and migration of SW/CDTK were attenuated to 50.41 and 80.12%, respectively (P<0.01), while those of SW/shCEACAM6-CDTK cells were reduced more significantly to 24.61 and 45.17%, respectively (P<0.01). By contrast, no differences were observed between SW1990 and SW/null (P>0.05) (Fig. 6B).

## Discussion

Mounting evidence suggests that combination cancer therapy has the potential to be effective in combating malignancies. Combination gene therapy has the advantages of gene therapy, elevates the therapeutic efficacy and overcomes the shortcomings of single gene therapy (18). Although suicide gene therapy is a potentially effective method for killing tumor cells *in vitro* and *in vivo*, the results of clinical trials indicate a need for greater efficacy (19). In previous studies, the co-transfer of vectors carrying different genes to enhance anti-tumor effect has been attempted (20). Combination of TK/CD gene therapy gene therapy with lipiodol embolism in the treatment of liver cancer may effectively inhibit cancer growth and prolong the survival time (9). In this study, to test the feasibility of a novel therapeutic vector system involving a combination of suicide and RNAi-based gene therapy, we initially constructed the novel vector pcDNA3.1(-)shCEACAM6-yCDglyTK, which was regulated by a U6 promoter, while the fusion suicide gene yCDglyTK was driven by a CMV-enhanced CEA promoter. Normal expression of each gene was confirmed by RT-PCR and western blot analysis. Pancreatic carcinoma cell lines stably expressing the CEACAM6 shRNA and yCDglyTK gene were then established and anti-tumor efficacy of the recombinant plasmid was evaluated *in vitro*.

Suicide genes are viral or bacterial enzymes capable of converting non-toxic prodrugs into toxic metabolites that cause tumor cell death when introduced into tumor sites. CD and HSV-TK are typical suicide genes that convert non-toxic prodrugs, such as 5-FC and GCV, into cytotoxic metabolites, such as 5-fluorouracil (5-FU) and GCV-TP, respectively (20–23). Combination of HSV-TK/GCV and CD/5-FC might have synergistic effects (24). Previous studies showed that yCD, a yeast-derived CD, is efficient at deaminating 5-FC to yield 5-FU, whereas bacterial CD (bCD) has a poor conversion efficiency (25). Moriuchi *et al*(26) reported that TK was able to mediate the phosphorylation of 5-FU metabolites, and might reduce the cytotoxicity of CD/5-FC system. GCV had the potential to interfere with 5-FC in the yCDglyTK system, thus 5-FC was used as the only prodrug in our study. Cytotoxicity of the yCDglyTK gene in the presence of prodrugs using MTT assay was tested. The mean cell viability decreased in a time-dependent manner in yCDglyTK- and shCEACAM6-yCDglyTK-transfected SW1990 cells, although not in untransfected SW1990 cells. Our results have shown that yCDglyTK was confirmed to be expressed in SW/CDTK and SW/shCEACAM6-CDTK cells, rendering the new system efficient in delivering suicide gene into cancer cells and inducing cytotoxicity.

CEACAM6 is a single-chain GPI-anchored immunoglobulin (Ig)-like glycoprotein and is a member of the human CEA family (27). Jantscheff *et al*(28) showed that CEACAM6 overexpression was associated with poor clinical outcome in colorectal cancer. CEACAM6 overexpression independently predicted poor overall survival and disease-free survival, whereas CEACAM1 or CEACAM5 was not significantly associated with these outcomes. CEACAM6 overexpression leads to morphology changes that are similar to epithelium-messenchymal-transformation (29), increased invasiveness (29), increased chemoresistance (30) and resistance to anoikis (31–33), whereas CEACAM6 appears to exert its pro-invasive effect in a c-Src-dependent manner, at least in part through the upregulation of MMP-9 activity (34). It has also been proposed that low levels of E-cadherin-mediated cell-to-cell interaction are important in tumor invasiveness and metastasis (35,36). Suppressing CEACAM6 gene expression or inhibiting CEACAM6 function can reverse these effects. Inhibition of CEACAM6 function using an antibody fragment can affect cell migration, invasion and adhesion *in vitro*(12,34). RNAi offers a unique opportunity to silence the expression of individual genes with a high degree of specificity, allowing the roles of individual genes to be dissected (37). In the present study, we investigated the invasion and migration-inhibitory effects of the yCDglyTK- and shCEACAM6-yCDglyTK-transfected SW1990 cells. The invasion and migration were significantly suppressed (P<0.01) in the two transfected groups. The inhibitory rates of shCEACAM6-yCDglyTK-transfected SW1990 cells were more prominent than those of yCDglyTK-transfected SW1990 cells. Suppression of the CEACAM6 transcripts using siRNA of CEACAM6 leading to a reduction in cancer cell invasiveness, may be associated with an increase in E-cadherin promoter activity (35). CEACAM6-siRNA or yCDglyTK alone has the potential to kill cancer cells and cause tumor growth delay, as well as inhibit tumor invasion and migration. However, a combination of the two genes has been shown to achieve a stronger anti-tumor effect, demonstrating a synergistic effect between CEACAM6-siRNA and yCDglyTK.

The novel recombinant plasmid was able to silence functional genome CEACAM6, inhibit tumor invasion and metastasis. However, there are potential defects with this new system. CEA protein only overexpresses in a majority of pancreatic cancer cells. In our triple-expressing vector pcDNA3.1(-) shCEACAM6-yCDglyTK, we used a CEA promoter to drive the expression of yCDglyTK, a treatment that specifically killed CEA-positive cancer cells. The novel shCEACAM6-yCDglyTK system had little effect on the CEA-negative pancreatic cancer cells. Moreover, a low level of CEACAM6 protein expression has been noted in a variety of normal human tissues, including granulocytes and epithelia from various organs (38) and this expression is also associated with infectious diseases (39,40). The novel system may therefore not only target specific tumor tissues. Strategies aiming to improve the safety of RNAi-based gene therapy are therefore necessary.

In conclusion, the results from the present study have demonstrated that CEACAM6-targeted RNAi with suicide gene therapies had a synergistic effect. Additionally, the combination gene therapy system may be a valid and viable strategy to inhibit the proliferation, as well as attenuate the invasiveness and metastasis of pancreatic carcinoma SW1990 cells *in vitro*. The present study provides a novel gene therapy strategy that is effective, not only for pancreatic cancer, but also for other CEACAM6-expressing tumors.

## Abbreviations:

CEACAM6: carcinoembryonic antigen-related cell adhesion molecule
CEA: carcinoembryonic antigen
shRNA: short hairpin RNA
GEPT: gene-direct enzyme/prodrug therapy
RNAi: RNA interference
CD: cytosine deaminase
HSV-TK: herpes simplex virus thymidine kinase
5-FC: 5-fluorocytosine
GCV: ganciclovir
